# Exploring the Therapeutic Potential of Bovine Colostrum for Cancer Therapies

**DOI:** 10.3390/ijms26167936

**Published:** 2025-08-17

**Authors:** Yalçın Mert Yalçıntaş, Mikhael Bechelany, Sercan Karav

**Affiliations:** 1 Department of Molecular Biology and Genetics, Çanakkale Onsekiz Mart University, Çanakkale 17000, Turkey; yalcinmertyalcintas@stu.comu.edu.tr; 2 Institut Européen des Membranes (IEM), Centre National de la Recherche Scientifique (CNRS), École Nationale Supérieure de Chimie de Montpellier (ENSCM), Unité Mixte de Recherche (UMR) 5635, University Montpellier, F-34095 Montpellier, France; 3 Functional Materials Group, Gulf University for Science and Technology (GUST), Masjid Al Aqsa Street, Mubarak Al-Abdullah 32093, Kuwait

**Keywords:** bovine colostrum, cancer, exosome, tumor, supplement

## Abstract

Colostrum is a nutrient-rich fluid secreted by mammals shortly after birth, primarily to provide passive immunity and support early immune development in newborns. Among its various sources, bovine colostrum is the most widely used supplement due to its high bioavailability, safety profile, and clinically supported health benefits. Rich in immunoglobulins, lactoferrin, growth factors, and antimicrobial peptides, bovine colostrum exhibits diverse biological activities that extend beyond neonatal health. Recently, the rising prevalence of cancer—driven by environmental stressors such as radiation, processed foods, and chronic inflammation, as well as non-environmental hereditary factors including germline mutations, family history, and epigenetic inheritance—has fueled interest in natural adjunctive therapies. Scientific studies have explored the anticancer potential of bovine colostrum, highlighting its ability to modulate immune responses, inhibit tumor growth, induce apoptosis in cancer cells, and reduce inflammation. Key components including lactoferrin and proline-rich peptides have been identified as contributors to these effects. Additionally, bovine colostrum may help reduce the side effects of standard cancer treatments, such as mouth sores from chemotherapy or weakened immune systems, by helping to heal tissues and boost the body’s defenses. While large-scale clinical studies are still needed, current findings suggest that bovine colostrum holds promise as a supportive element in integrative cancer care. In conclusion, bovine colostrum represents a safe, bioactive-rich natural supplement with multifaceted therapeutic potential, particularly in oncology, owing to its key components such as lactoferrin, immunoglobulins, growth factors (e.g., IGF-1, TGF-β), and proline-rich polypeptides (PRPs), which contribute to its immunomodulatory, anti-inflammatory, and potential anticancer effects. Ongoing and future research will be crucial to fully understand its mechanisms of action and establish its role in evidence-based cancer prevention and treatment strategies.

## 1. Introduction

**Figure 1:**
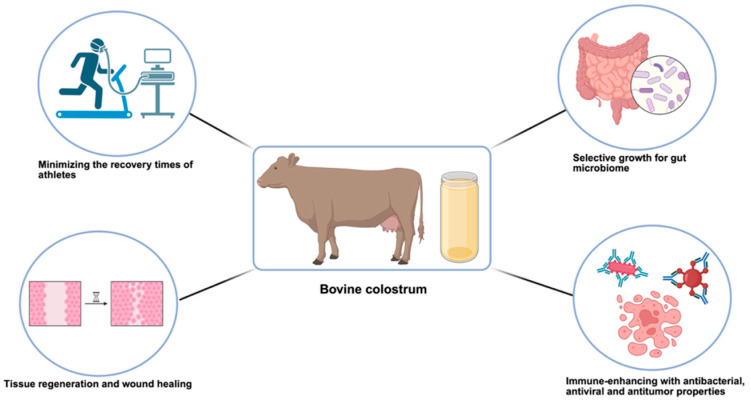
Therapeutic effects provided to the body by bovine colostrum supplementation. Bovine colostrum supplementation promotes selective growth on the gut microbiome due to its oligosaccharides, has a positive effect on tissue regeneration and recovery time through growth hormones, and exhibits antimicrobial and antiviral properties through its bioactive components (lactoperoxidase, lactoferrin, immunoglobulins) [2,4,10].

Bovine colostrum is a constructive and highly supportive supplement, secreted by mammals for a specific period after birth [1]. Although mature bovine milk is more abundant and economically accessible, bovine colostrum is preferred in therapeutic applications due to its significantly higher concentrations of bioactive compounds—including immunoglobulins, lactoferrin, growth factors, oligosaccharides, and antimicrobial peptides—that are either absent or present at much lower levels in mature milk [1]. These components contribute not only to immune and cognitive development in newborns, but also hold therapeutic potential for various diseases, as summarized in Table 1 and illustrated in Figure 1 [2,3,4]. These include gastrointestinal disorders (e.g., inflammatory bowel disease, diarrhea), infections (bacterial and viral), immune-related conditions, and even certain cancers. In recent years, growing interest in these components has led to a significant increase in research focused on their immunomodulatory, antimicrobial, and regenerative properties. Notable features of bovine colostrum include its ability to enhance immune function—primarily through the action of immunoglobulins (especially IgG), which provide passive immunity by neutralizing pathogens; lactoferrin, which exhibits immunomodulatory and anti-inflammatory effects [3]; and proline-rich polypeptides (PRPs), which help regulate immune cell activity. Additionally, colostrum-derived cytokines and growth factors such as Transforming Growth Factor-β (TGF-β) contribute to mucosal immunity and the development of immune tolerance [2,4].

**Table 1:** Concentrations and health implications of bovine colostrum components.

Component	Health Implication	Concentration	Reference
Lactoferrin	Supports immune system, has antimicrobial properties, and may aid in iron regulation and gut health.	0.82 mg/mL	[2,5,6]
Immunoglobulins	Help protect the body by identifying and neutralizing pathogens, playing a key role in immune defense.	IgG: 46.40 g/L IgM: 6.77 g/L IgA: 5.86 g/L	[7]
Exosomes	Support immune development, carry regulatory molecules like miRNAs, and may serve as biomarkers for health and disease.	-	[8]
Oligosaccharides	Support gut health, promote beneficial microbes, and enhance immune protection in early life.	--	[5,9]
Lactoperoxidase	Supports immune defense by exhibiting antimicrobial activity against bacteria, viruses, and fungi.	-	[2,3]

Cancer is one of the most dangerous issues. As the second leading cause of mortality, cancer presents major problems for public health and necessitates continuous research efforts to improve early diagnosis, therapeutic efficacy, and patient outcomes [11]. The mechanism of cancer is based on the uncontrollable division of cells. Cancer cells continue to divide, leading to the formation of tumors. Radiation and genetic mutations, among other factors, can contribute to this malfunction. The complexity of cancer arises from its heterogeneous nature, with various types and subtypes exhibiting distinct biological behaviors, treatment responses, and prognoses. Methods have been developed, but despite the existence of highly effective treatments, a complete solution has not been produced [12]. Cancer usually starts with low symptoms, which makes early diagnosis difficult. Although the methods used to stop the progression of cancer are effective, early diagnosis is crucial for preventing the disease [13].

Although genetic abnormalities can cause cancer, environmental factors can also cause cancer to develop in healthy people. These environmental factors could include radiation exposure, air pollution, and carcinogenic chemicals present in commonplace items. Although several factors might contribute to the development of cancer, it can be argued that environmental factors may negatively affect people who are genetically susceptible to the disease [14,15]. Cancer is a complex disease involving the deregulation of multiple signaling pathways, and hundreds of genes have been identified to play a role in tumor development. Therefore, the mechanism of cancer formation can vary in many ways. Studies have found approximately 300 gene mutations that can cause cancer, representing more than 1% of the human genome [16]. Cancer, resulting from disruptions in cell growth and proliferation processes, generally arises due to alteration in signaling pathways. When certain genes, known as oncogenes, become overly active or are stimulated by mutations, they promote cell division. During this growth process, if tumor suppressor genes are inhibited or fail to intervene due to similar alterations, uncontrolled cell proliferation occurs. Mutations are considered the primary cause of these issues, and various environmental or hereditary factors can lead to mutations. When mutations disrupt the intracellular signaling pathways, the cell begins uncontrolled proliferation, evades immune detection, and spreads to other parts of the body (metastasis), initiating the overall cancer mechanism [17,18]. This process is highly complex and supports the elevated heterogenecity of tumors.

In addition to conventional cancer treatments—such as chemotherapy, radiotherapy, and immunotherapy, which are often associated with side effects like immunosuppression, gastrointestinal toxicity, and fatigue—nutritional supplementation can play a supportive role in improving patient outcomes [12]. However, because certain supplements may interact with treatment pathways or immune responses, cancer patients must be cautious and follow selective, evidence-based dietary strategies. Bovine colostrum supplementation, as a treatment for many diseases, has potential therapeutic effects against cancer [19,20]. This review article examines the potential therapeutic effects of bovine colostrum on cancer and evaluates the clinical studies conducted in this field, along with their outcomes.

## 2. Exploring Cancer: Mechanisms, Types, and Statistics

**Figure 2:**
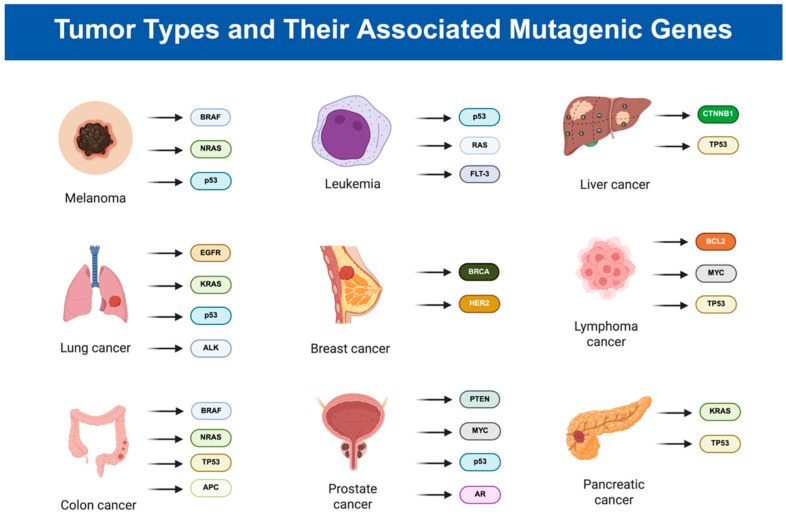
Tumor types and their associated mutagenic genes.

Cancer can occur in different parts of the body and is usually named after the cells of origin. Based on recorded data, the major types of cancer have been examined [21]. Breast cancer, common in women, usually begins in the milk ducts or lobules and is associated with (Breast Cancer Gene) BRCA gene mutations, hormonal imbalances, and overexpression of Human Epidermal Growth Factor Receptor 2 (HER2) (Figure 2) [22], with over 300,000 cases and 40,000 deaths presented in 2024 [23]. Colorectal cancer (CRC) includes colon and rectal cancers and involves mutations in Adenomatous Polyposis Coli (APC), KRAS, B-Raf Proto-Oncogene, Serine/Threonine Kinase (BRAF), and Tumor Protein 53 (TP53) genes (Figure 2), often progressing silently until diagnosed [24,25]; over 150,000 cases and 50,000 deaths are projected in 2024 [26]. Lung cancer, strongly linked to tobacco use, is divided into Small Cell Lung Cancer (SCLC) and Non-Small Cell Lung Cancer (NSCLC), with mutations in Epidermal Growth Factor Receptor (EGFR), KRAS, Anaplastic Lymphoma Kinase (ALK), p53, and RB (Figure 2) playing key roles [27,28]; it is expected to cause over 125,000 deaths from 230,000 cases in 2024 [23,26]. Leukemia arises from mutations in bone marrow stem cells, leading to the accumulation of immature blood cells and disruption of normal blood cell production. It has four types—Acute Lymphoblastic Leukemia (ALL), Acute Myeloid Leukemia (AML), CLL (Chronic Lymphocytic Leukemia), and Chronic Myeloid Leukemia (CML)—each involving different gene mutations [29,30]. Over 60,000 leukemia cases and 20,000 deaths are projected for 2024 [23].

Prostate cancer, common in men and increasing with age, results from the uncontrolled proliferation of cells in the prostate gland due to genetic mutations and hormonal imbalances. Key genes involved include Phosphatase and Tensin Homolog (PTEN), Myelocytomatosis Viral Oncogene Homolog (MYC), p53, and Androgen Receptor (AR) (Figure 2), with mutations causing uncontrolled cell growth and resistance to apoptosis [31,32]. Hormonal imbalances, particularly involving testosterone, contribute to the disease’s progression. Prostate cancer often shows no symptoms in early stages but can lead to urinary issues, pain, and metastasis as it advances. Regular check-ups are crucial for early detection, as prostate cancer is expected to account for around 300,000 cases and 35,000 deaths in 2024 [23]. Melanoma, originating from melanocytes, is caused by excessive UV exposure, leading to mutations in genes such as BRAF, Neuroblastoma RAS Viral Oncogene Homolog (NRAS), and p53 (Figure 2), which promote uncontrolled cell proliferation. The cancer has high metastatic potential, spreading to vital organs if not detected early. Symptoms include changes in moles, itching, and ulceration, with an expected 100,000 cases and over 8000 deaths in 2024 [23]. Liver cancer, including primary hepatocellular carcinoma (HCC), is linked to chronic liver conditions and genetic mutations including TP53 and Catenin Beta-1 (β-catenin) (CTNNB1) (Figure 2) [33]. Secondary liver cancer, caused by metastasis from other cancers, also affects the liver. Symptoms such as abdominal pain, jaundice, and fatigue appear in later stages, with over 40,000 cases and 29,000 deaths expected in 2024 [23,34]. Pancreatic cancer, linked to smoking, chronic pancreatitis, and obesity, often progresses silently and is associated with mutations in genes including KRAS and TP53 (Figure 2) [35]. Symptoms include jaundice, weight loss, and abdominal pain, with over 65,000 cases and 50,000 deaths expected in 2024, giving it a high mortality rate of 78% [23,36]. Lymphoma, a cancer of the lymphatic system, involves mutations in genes such as B-cell Lymphoma 2 (BCL2), MYC, and TP53 (Figure 2), leading to abnormal lymphocyte proliferation [37,38]. Symptoms include swollen lymph nodes, fatigue, and weight loss, with over 80,000 cases and 20,000 deaths expected in 2024 [23]. Early diagnosis and treatment are crucial for improving prognosis in all these cancers.

## 3. Bovine Colostrum as Therapeutic Agent

Colostrum has become a widely used supplementary material. Especially when considering its use in various diseases, it has demonstrated a variety of therapeutic effects due to the bioactive components it contains [39]. Bovine colostrum supplementation is highly important for humans because the components produced by cows are particularly effective on the human body, especially when considering immunomodulatory factors [2,40]. Additionally, when it comes to production, bovine colostrum is both economical and beneficial due to its high yield and concentration of bioactive components [41,42]. Although studies directly examining the relationship between bovine colostrum and cancer are currently limited, existing evidence suggests its promising potential as a complementary strategy in oncology. Bovine colostrum supplementation has gained global attention for its therapeutic applications, both as a standalone treatment and as an adjunct. Notably, it has shown efficacy in managing gastrointestinal disorders (e.g., infectious diarrhea, inflammatory bowel disease), respiratory tract infections, immune deficiencies, wound healing, and cancer-related complications. Notably, its high concentration of oligosaccharides, which benefit the microbiome, its glycoproteins that promote selective microbial growth, its immune-supporting agents that protect against diseases or enhance resilience during illness, and its bioactive growth hormones that contribute to the body’s anabolic processes all highlight its multifaceted benefits [2,4,9,43]. Additionally, for professional athletes, bovine colostrum aids in reducing recovery time and supporting bodily renewal after intense training sessions [10]. These properties serve as evidence of its effectiveness as a supplement across diverse fields.

Additionally, bovine colostrum is rich in oligosaccharides and glycoprotein concentrations, which further support its therapeutic effects against cancer through supplementation [44]. Prebiotics have therapeutic effects against cancer and hold promising potential for future treatments. By selectively promoting the growth of gut microbiota, they increase the production of short-chain fatty acids (SCFAs) and reduce the concentration of harmful cancer-related components (such as secondary bile acids) [43,45]. Studies have shown that β (1–4) galacto-oligosaccharides (GOS) and fructo-oligosaccharides have the ability to reduce colorectal cancer (CRC) risk [45]. Plant-derived pectin oligosaccharides (POS) and novel galacto-oligosaccharides (GalOS) regulate oxidative and inflammatory pathways (such as AMP-Activated Protein Kinase (AMPK) and Nuclear Factor kappa-light-chain-enhancer of activated B cells (NF-κB)), exhibiting antioxidant and anti-inflammatory effects [46,47]. These effects have the potential to trigger apoptosis in tumor cells and inhibit tumor growth. However, further research is required on this topic. Additionally, a study has reported that chitosan oligosaccharides (COS) suppressed tumor growth in lung cancer models and enhanced immune responses, providing positive effects on the body [48]. The changes in glycans present in the body during the cancer process can provide significant insights into the disease. For instance, in breast cancer, the increase in Lewis antigens and branched *N*-glycans on the cell surface facilitates tumor cells’ evasion of the immune system and directly impacts the metastasis process [49]. In CRC cancer, changes observed in glycans lead to metastasis by enhancing the ability of cancer cells to adhere to other tissues [50,51]. In another type of cancer, liver cancer, specific glycoforms such as AFP-L3 are promising biomarkers for early diagnosis and are important for predicting disease prognosis [52]. In summary, glycans can serve as biomarkers providing information about cancer and, through their use by malignant tumors, may occasionally deceive the immune system. Among its many advantages, these mentioned benefits increase the use of bovine colostrum compared to other colostrum sources. Bovine colostrum supplementation can be available in various forms, and the processes involved in achieving these forms may affect its bioactivity [53]. In addition to their proposed therapeutic effects, bovine colostrum and bovine milk have been evaluated in several experimental and clinical studies targeting cancer. While bovine colostrum has shown promising immunomodulatory and antiproliferative effects, some clinical outcomes also derive from bovine milk or milk-derived components. These findings are summarized in Table 2. Given the scarcity of current studies, further experimental and clinical investigations are necessary to validate the therapeutic relevance of bovine colostrum in cancer treatment.

The clinical studies on bovine colostrum, particularly applied to cancer types, are presented in Table 2. Upon examining these studies, the results appear to be quite impressive. Lactoferrin-based supplementation methods have demonstrated therapeutic effects on tumor development and achieved inhibition [70]. In some studies, this efficacy was enhanced through combination with lactoperoxidase, reporting a significant reduction in tumor growth. These findings present promising results for cancer treatment. Additionally, the different forms of lactoferrin, holo- and apo-, have been utilized in supplementation, showing effective outcomes in preventing tumor cell metastasis and invasion [71,72]. These forms also play a regulatory role in the expression of epithelial and mesenchymal proteins, which are critical in tumor growth and metastasis. When these findings are evaluated, it is evident that lactoferrin is a clinically validated therapeutic agent, and further research is anticipated to uncover additional potential therapeutic effects.

**Figure 3:**
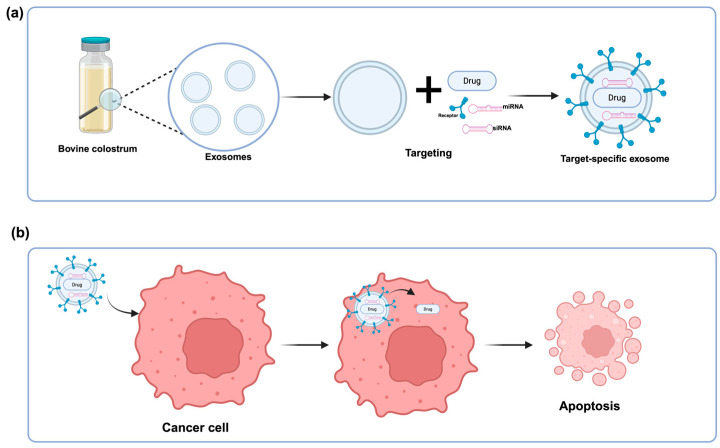
Target-specific regulation of exosomes and the process of tumor cell death. (**a**) Exosomes isolated from bovine colostrum are designed with receptors, miRNAs, chemotherapy drugs and siRNAs to make them target specific. (**b**) Target-specific exosomes deliver the drug they carry to the target tumor cell, and as a result of this process, programmed cell death is triggered in the tumor cell [65,73].

In addition to traditional approaches, the table also presents studies utilizing the innovative method of bovine colostrum-based exosomes for drug delivery, which have shown promising results in targeted and immunotherapy [73]. Exosomes are extracellular vesicles involved in intercellular communication by transporting cell-based components such as proteins, lipids, and RNA [74]. As natural nanoparticles, exosomes have gained significant attention in biotechnological and biomedical research due to their ability to deliver therapeutic molecules and target specific cells or tissues. Particularly, the use of bovine milk-derived exosomes in studies offers advantages such as greater stability, efficiency, and the ability to effectively deliver microRNAs and other therapeutic molecules to target cells, thereby enhancing their potential in treating various diseases [75] (Figure 3).

Bovine colostrum is a supplement rich in bioactive components, and exosomes isolated from bovine colostrum have gained priority in therapeutic applications due to their high concentration, biocompatibility, and immune system compatibility [1,75,76]. These vesicles are designed for the transport and protection of bioactive molecules, making them highly suitable candidates for drug and therapy-based encapsulation processes. Their stability under various conditions and ease of isolation further highlight the advantages of bovine-derived vesicles [75,76]. When clinical studies were examined, colostrum-based exosome drug delivery treatments significantly reduced the presence of tumor cells in a short period (1–2 days) [62,74]. These exosomes have shown promising results in cancer treatment through various mechanisms such as cytotoxicity, generation of reactive oxygen species (ROS), and inhibition of cancer cell migration [62,74]. Additionally, when specifically used on lung cancer tumor cells, they inhibited tumor growth and significantly reduced toxicity [65]. In addition to their cytotoxic effects, colostrum-based exosome therapy has reduced primary tumor growth in colorectal and breast cancers. Targeting cancer-specific mutagenic genes such as KRAS and p53 has inhibited lung tumor growth, limited KRAS gene expression, regulated p53 expression, and enhanced chemo-sensitization to paclitaxel [66,67].

The impact of bovine colostrum supplementation on potential complications following cancer treatment has also been investigated. As shown in Table 3, using bovine colostrum as a supplement after cancer treatments has shown various therapeutic effects. Considering this, bovine colostrum supplementation is highly significant for both ongoing treatment and post-treatment phases.

Previous studies have reported that bovine colostrum supplementation can minimize the physiological side effects caused by chemotherapy [80]. These beneficial effects are mainly attributed to its rich composition of bioactive peptides, immunoglobulins, and growth factors, which contribute to enhancing tissue repair and supporting immune homeostasis. Moreover, bovine colostrum has been shown to reduce inflammation and alleviate symptoms associated with chemotherapy-induced gastrointestinal disorders [81]. Such findings suggest that colostrum may act not only as a supportive nutritional intervention but also as a functional therapeutic agent that mitigates treatment-related toxicity. By improving patients’ tolerance to chemotherapy and reducing adverse events, bovine colostrum supplementation may ultimately contribute to better treatment adherence, enhanced recovery, and improved quality of life in cancer patients.

## 4. Future Perspective

The standardization process is particularly important for dietary supplements. Ensuring that the ingredients used meet defined standards plays a critical role in addressing health concerns and establishing global reliability. In this context, the standardization of bovine colostrum is essential to ensure the consistency of quality and content in colostrum-based supplements. Standardization aims to regulate the levels of bioactive compounds found in colostrum—such as immunoglobulins, growth factors, and lactoferrin—so that they are present in the desired concentrations. This process can vary depending on multiple factors, including the breed of the cow, duration of lactation, nutrition, and overall health status. Typically, colostrum is collected within the first few hours after birth, then tested for active compound levels and purity, and subsequently processed using low-temperature drying methods to preserve biological activity. During this process, manufacturers utilize various molecular biology and analytical chemistry techniques to measure active ingredients. Based on the results, compliance with quality protocols such as ISO certification and Good Manufacturing Practices (GMP) is assessed. Through proper standardization, bovine colostrum can be ensured as a safe, effective, and consistent functional food or dietary supplement. Establishing clear standardization processes for colostrum and enhancing the reliability of its active components have the potential to significantly increase the production and consumption of colostrum-based functional foods and supplements in the future.

The consumption and supplementation of bovine colostrum have become increasingly common in the management of various diseases due to its rich content of bioactive components. This review highlights the therapeutic potential of these components across multiple domains, including cancer. Over time, research has uncovered new functionalities of colostrum-derived molecules, particularly exosomes, which may offer targeted and safer treatment options by modulating tumor-related pathways. However, it is important to consider administration routes and delivery limitations. While colostrum is generally consumed orally as a dietary supplement, the clinical use of its purified components—such as exosomes or peptides—may require alternative delivery methods (e.g., intravenous or local injection) to ensure bioavailability and therapeutic efficacy. Therefore, further studies are needed to determine optimal dosages, delivery strategies, and safety profiles in cancer therapy. Notably, scientific interest in the relationship between colostrum and cancer has remained steady over the past decade. This consistency reflects the evolving evidence supporting its multi-faceted therapeutic roles in different cancer types. The expanding scope of related research suggests that bovine colostrum could be evaluated from diverse biomedical perspectives, offering potential for novel treatment strategies in the future.

## 5. Conclusions

Colostrum is the initial milk-like secretion produced by mammals during the early postpartum period and plays a vital role in transferring passive immunity from the mother to the newborn. Rich in immunoglobulins, growth factors, antimicrobial peptides, and various bioactive molecules, colostrum is essential for the development of the neonatal immune system and intestinal health. Beyond its primary physiological function, colostrum has attracted increasing attention in recent years for its wide-ranging therapeutic potential, supported by a growing body of preclinical and clinical research.

Cancer, defined as a group of diseases characterized by the uncontrolled proliferation, invasion, and sometimes metastasis of abnormal cells, remains a major global health burden. Despite advances in conventional treatment approaches such as surgery, chemotherapy, radiotherapy, and immunotherapy, challenges like drug resistance, systemic toxicity, and limited specificity continue to drive the need for novel and safer therapeutic alternatives. In this context, naturally derived compounds with multi-functional biological properties are of particular interest. Several studies have demonstrated that bovine colostrum in particular contains components with potential anticancer properties. Among these, lactoferrin has been shown to inhibit tumor initiation and progression in gastric cancer models, modulate the tumor microenvironment, and interfere with key signaling pathways involved in cancer cell proliferation and survival. Additionally, lactoferrin has been implicated in the regulation of iron metabolism, oxidative stress, and immune cell activation, all of which are relevant to tumor biology. Other colostrum-derived molecules, such as transforming growth factors and immunoglobulins, may also exert modulatory effects on cancer cell behavior. A particularly promising area of recent research is the study of colostrum-derived extracellular vesicles, especially exosomes. These nanosized vesicles serve as natural carriers of proteins, lipids, and nucleic acids, and have been explored for their potential use in targeted drug delivery systems. In various experimental models, bovine colostrum-derived exosomes have successfully delivered therapeutic agents, resulting in enhanced anticancer activity. Notable findings include increased cytotoxicity, reactive oxygen species (ROS) production, inhibition of cancer cell migration, and a significant reduction in tumor growth, particularly in lung cancer models. Moreover, these exosomes demonstrated superior safety profiles compared to traditional chemotherapeutics, with reduced systemic toxicity. Mechanistically, some studies have reported their involvement in downregulating KRAS expression, restoring p53 activity, and sensitizing tumors to standard drugs like paclitaxel.

As a result, these findings highlight bovine colostrum as a valuable source of bioactive compounds with the potential to contribute to cancer prevention and therapy. However, translating these experimental results into clinical applications requires further investigation. Comprehensive mechanistic studies, rigorous clinical trials, and the development of standardized formulations are essential steps in realizing colostrum’s full therapeutic promise. As research continues to evolve, colostrum may emerge not only as a functional food supplement but also as a complementary tool in the broader landscape of cancer treatment.

## Figures and Tables

**Table 2:** Clinical uses of bovine colostrum supplementation against different cancer types.

Cancer Type	Study Design	Target Group	Supplement and TARGET	Dose and Duration	Effect	Reference
Esophagus cancer	in vitro experimental study	Esophageal cancer cell line KYSE-30	Lactoferrin from bovine colostrum	500 μg/mL for 20 h	Lactoferrin has blocked the growth of tumor cells.	[54]
Tumor cells	in vitro comparative study	Human cancer cell lines HepG-2, Caco-2, MCF-7 and PC-3	Nanoparticle-based Lactoperoxidase and Lactoferrin	315–1388 μg/mL for 72 h	Nanoparticle-based compounds have inhibited tumor development and growth.	[55]
Lung cancer	in vivo experimental study	5-week-old F344 rats (weighing 70–90 g)	Liposomal bovine lactoferrin	≥10 μg/mL for 8 weeks	Tumor development and uncontrolled proliferation have been inhibited.	[56]
Colorectal cancer	ex vivo observational study	Tumor-derived cells from CRC patients	KMP01D	10 mg KMP01D and 0.025 μg vitamin D3/5 for 24 h	Demonstrated anti-inflammatory effects in immune cells from CRC patients by reducing inflammatory cytokines and enhancing apoptosis.	[57]
Lung cancer	combined in vitro and in vivo experimental study	Human lung adenocarcinoma cell lines A549	Lactoferrin	15 to approximately 1 mg/mL for 48 h	Inhibited lung cancer by reducing the expression of vascular endothelial growth factor (VEGF) and inflammatory cytokines (TNF-α, IL-4, IL-6, IL-10), thereby limiting tumor growth and inflammation in both cell and animal models.	[58]
Gastric cancer	in vitro experimental study	Stomach cancer cell line, HEK-293 and HFF	Lactoferrin	500 µg/mL for 20, 36, 48 h	Lactoferrin inhibited stomach tumor cells.	[59]
Ovarian cancer	in vitro mechanistic study	Ovarian cancer cell lines SKOV-3 and A2780	Bovine serum	The treatment duration varied and was terminated at 24, 48, 72, and 96 h.	Inhibited the proliferation, migration, and invasion of ovarian cancer cells.	[60]
Lung cancer	in vitro and in vivo mechanistic study	A549 lung cancer cells in vitro and immunocompromised mice bearing subcutaneous and orthotopic lung tumors	siNRF2 delivered via bovine colostrum-derived exosome–polyethyleneimine matrix (EPM)	20 μg	Significant reduction of tumor growth.	[61]
Breast cancer	in vitro experimental study	T-47D, MDA-MB-231, and BT-474 breast cancer cells	Bovine milk exosomes	500 µg/mL for 68–70 h	Drug delivery was achieved through bovine exosomes, leading to the inhibition of cancer growth.	[62]
Tumor cells	in vitro evaluation	C6 rat glioblastoma cell line.	Bovine colostrum peptide	312.7 ± 3.5 mg/mL for 48 h	Reduced tumor cell population by 50% after 48 h.	[63]
Breast cancer	in vitro experimental study	The MDA-MB-231 (MD Anderson Metastatic Breast Cancer) cell lines	Bovine milk-derived exosomes loaded with dihydroartemisinin	24, 48, and 72 h	Enhanced its anticancer activity, including cytotoxicity, ROS generation, and inhibition of migration, improving its efficacy against cancer cells.	[64]
Lung cancer	in vitro and in vivo experimental designs	Human lung cancer cell lines A549 and mice bearing subcutaneous and orthotopic lung tumors	Bovine colostrum-derived exosomes and paclitaxel	6 mg/kg (low dose) or 4 → 8 mg/kg (escalated dose) for 7 weeks	Significantly inhibited lung cancer growth and reduced toxicity compared to conventional treatments	[65]
Colorectal and breast cancer	in vitro and in vivo experimental designs	4T1.2, LIM1215 and MCF7 cells	Bovine milk-derived extracellular vesicles	100 µg/mL in vitro (for 48–72 h) and 25 mg/kg orally in vivo	Components of bovine colostrum reduced primary tumor growth in colorectal and breast cancer but accelerated metastasis, with timing of administration influencing these effects.	[66]
Many cancer types	in vitro and in vivo experimental designs	Lung: H1299, A549, H522; pancreatic: Panc-1, MiaPaCa-2; breast: MDA-MB-231 cell lines	Bovine colostrum-derived exosome–polyethyleneimine matrices (EPM)	0.01–20 μg for 48 h	Targeting KRAS and p53 inhibited lung tumor growth, reduced KRAS expression, restored p53 expression, and enhanced chemo-sensitization to paclitaxel.	[67]
Breast cancer	in vitro experimental design	Four triple-negative breast cancer (TNBC) cell lines (MDA-MB-231, MDA-MB-468, HCC1806, HCC1937)	Bovine milk-derived exosomes	20 μM for 48 h	Reported a 50% reduction in cell viability.	[68]
Breast cancer	in vitro experimental design	Human breast cancer cells MDA-MB-231 and MCF-7	Bovine Apo- and Holo- lactoferrin	Durations ranging from 20 min to 48 h	Inhibited migration and invasion, and modulated expression of epithelial and mesenchymal proteins.	[69]

**Table 3:** Colostrum supplementation after the cancer therapies.

Disease	Study Design	Supplement	Effect	Reference
Gut toxicity	in vivo experimental design, orally fed daily for 10–11 days.	Bovine colostrum	Reduce gut toxicity during chemotherapy by preserving intestinal function and reducing inflammation.	[77]
Gastrointestinal Toxicity	A randomized, double-Blind, Placebo-Controlled, daily oral supplementation for 4 weeks during induction therapy.	Bovine colostrum	Reduced the severity of oral mucositis compared to placebo in cancer patients.	[78]
Oral mucositis	in vivo experimental design, 5 days orally by gavage (either pre- or post-OM induction, depending on group)	Bovine colostrum	No significant effect of bovine colostrum on the healing of oral mucositis was observed.	[79]
Chemotherapy-induced physiological parameters	in vivo experimental design, orally administered daily for 4 weeks at doses of 500, 1000, or 1500 IU/kg	Bovine colostrum	Colostrum improved physiological, immune, and circulatory functions while reducing the negative effects of Etoposide.	[80]
Intestinal toxicity	in vivo experimental design, orally fed for 5 days	Bovine colostrum	Reduced gastrointestinal toxicity and inflammation.	[81]
Gastrointestinal mucositis	in vivo experimental design, 250 or 500 mg/kg Bovine colostrum-derived immunoglobulin gavaged twice daily for 10 days	Bovine serum-derived immunoglobulin	Reduced the incidence, severity, and duration of irinotecan-induced mucositis and gastrointestinal damage.	[82]
Neutropenia	in vivo experimental design, 20 g/day COL supplementation for 4 weeks	Bovine colostrum	Increase absolute neutrophil counts in patients with ALL undergoing chemotherapy.	[83]

## Data Availability

Not applicable. This article is a review; no new data were generated or analyzed. All data discussed are from previously published studies as cited.

## Abbreviations

AFP-L3: Alpha-Fetoprotein-L3, ALK: Anaplastic Lymphoma Kinase, ALL: Acute Lymphoblastic Leukemia, AML: Acute Myeloid Leukemia, AMPK: AMP-Activated Protein Kinase, APC: Adenomatous Polyposis Coli, AR: Androgen Receptor, BCL2: B-cell Lymphoma 2, BRAF: B-Raf Proto-Oncogene, BRCA: Breast Cancer Gene, CLL: Chronic Lymphocytic Leukemia, CML: Chronic Myeloid Leukemia, COS: Chitosan Oligosaccharides, CRC: Colorectal Cancer, CTNNB1: Catenin Beta-1, EGFR: Epidermal Growth Factor Receptor, GalOS: Galacto-Oligosaccharides, GMP: Good Manufacturing Practices, GOS: Galacto-Oligosaccharides, HER2: Human Epidermal Growth Factor Receptor 2, ISO: International Organization for Standardization, KRAS: Kirsten Rat Sarcoma Viral Oncogene Homolog, MYC: Myelocytomatosis Viral Oncogene, NF-κB: Nuclear Factor kappa-light-chain-enhancer of activated B cells, POS: Pectin Oligosaccharides, PTEN: Phosphatase and Tensin Homolog, RB: Retinoblastoma Protein, ROS: Reactive Oxygen Species, SCFAs: Short-Chain Fatty Acids, siRNA: Small Interfering RNA, TP53: Tumor Protein p53.
